# A Retrospective Chart Review Study on the Burden of Illness of Acid Sphingomyelinase Deficiency in Brazil

**DOI:** 10.3390/jcm15020589

**Published:** 2026-01-12

**Authors:** Roberto Giugliani, Ana Cecília Menezes de Siqueira, Ana Maria Martins, Bianca Fernandes Marcondes, Carolina Fischinger Moura de Souza, Dafne Dain Gandelman Horovitz, Emília Katiane Embiruçu Leão, Gaelle Gusto, Gerson da Silva Carvalho, Osvaldo Artigalás, Raquel Boy, Rodrigo Rosa de Stefani, Neeraj Singh Rawat, Gerasimos Konidaris

**Affiliations:** 1 Postgraduate Program in Genetics and Molecular Biology, Federal University of Rio Grande do Sul (UFRGS), Medical Genetics Service, Hospital de Clínicas de Porto Alegre (HCPA), Instituto Nacional de Ciência e Tecnologia em Multiômica Aplicada à Saúde de Precisão (IMASP), Dasa Genomics and Casa dos Raros, Porto Alegre 90035-903, Brazil; 2 Treatment Center of Inborn Errors of Metabolism, Instituto de Medicina Integral Professor Fernando Figueira, Recife 50070-902, Brazil; 3 Reference Center in Inborn Errors of Metabolism, Federal University of São Paulo, São Paulo 04021-001, Brazil; 4 Sanofi, São Paulo 04794-000, Brazil; 5 Clinical Research Servicer, Casa dos Raros, Hospital de Clínicas de Porto Alegre (HCPA), Porto Alegre 90035-903, Brazil; 6 Centro de Genetica Medica, Instituto Nacional de Saude da Mulher, da Criança e do Adolescente Fernandes Figueira, Fundação Oswaldo Cruz, Rio de Janeiro 22250-020, Brazil; 7 Department of Medicine, University of Bahia State, Salvador 41150-000, Brazil; 8 Certara, 7500 Paris, France; 9 Hospital de Base do Distrito Federal Hospital da Criança de Brasília, Brasília 70684-831, Brazil; 10 Medical Genetics Service, Moinhos de Vento Hospital, Porto Alegre 90560-032, Brazil; 11 Medical Genetics, Pedro Ernesto University Hospital—UERJ, Rio de Janeiro 20551-030, Brazil; 12 Medical Genetics Service, Hospital de Clínicas de Porto Alegre (HCPA), Porto Alegre 90035-903, Brazil; 13 Sanofi, Hyderabad 500081, India; 14 Sanofi, Reading RG6 1PT, UK

**Keywords:** acid sphingomyelinase deficiency, disease burden, Brazil, Hospital de Clínicas de Porto Alegre, retrospective study, manifestations, healthcare resource utilization

## Abstract

**Background**: Acid sphingomyelinase deficiency (ASMD) is a rare, progressive lysosomal storage disease with heterogeneous clinical manifestations. Evidence on the disease burden of ASMD is limited in Brazil. **Methods**: This observational, multicenter, retrospective study assessed the characteristics and clinical data of patients with ASMD type B and type A/B. Patients’ demographic data were retrieved from Hospital de Clínicas de Porto Alegre between January 1, 1986 and May 31, 2021, and available medical records were collected from eight centers in Brazil. **Results**: The study included 124 patients (full cohort: ASMD type B [75.8%] and type A/B [24.2%]; median [interquartile range {IQR}] age: 10.0 [3.6–19.9] years at diagnosis, *n* = 94), while medical records were available for 24 patients (subset cohort: ASMD type B [87.5%] and type A/B [12.5%]; median [IQR] age: 6.7 [1.9–11.3] years at diagnosis). Hepatobiliary and splenic manifestations were the most common clinical findings at symptom onset/diagnosis (75.0% and 70.8%, respectively) and at the last follow-up/death (83.3% each), with the majority of patients showing abnormal liver function parameters at both time points. At least 50.0% of patients had comorbidities at symptom onset or diagnosis. The incidence of hospitalization was reported in 33.3% patients at symptom onset/diagnosis and in 45.9% at the last follow-up/death. During the follow-up period, two patients with ASMD type A/B died in the subset cohort. **Conclusions**: The study provides insights into the high burden of illness in patients with ASMD, highlighting the need for disease awareness and early diagnosis in Brazil.

## 1. Introduction

Acid sphingomyelinase deficiency (ASMD), known as Niemann–Pick disease type A (OMIM: #257200), type A/B, and type B (OMIM: #607616), is a rare, autosomal recessive, lysosomal storage disease, caused by pathogenic variants in the sphingomyelin phosphodiesterase 1 (*SMPD1*) gene, resulting in a deficiency of the acid sphingomyelinase enzyme (ASM; EC 3.1.4.12) [1,2]. This leads to the progressive accumulation of sphingomyelin and other metabolically related lipids in monocytes, macrophages, and hepatocytes, primarily impairing functions of the spleen, liver, and lungs, as well as the central nervous system in severe phenotypes [1,3,4]. ASMD presents a heterogeneous phenotypic spectrum and is categorized into three subtypes; type A (infantile neurovisceral) constitutes approximately 30.0% of cases with early onset in infancy, rapidly progressive visceral manifestations, and neurodegeneration, leading to death by the age of 3 years [3]; type A/B (chronic neurovisceral) is defined by slowly progressive neurodegeneration and prolonged survival; and type B (chronic visceral) [2,3] is characterized by onset between infancy and adulthood [5] with multisystemic manifestations [1,2,4].

The estimated global prevalence of ASMD is 0.4–0.6 per 100,000 live births [6,7,8,9,10]. A reference laboratory determined the prevalence of ASMD (type A, type A/B, and type B) to be 0.33 per 100,000 live births in Brazil (2000–2013) [11]. However, ASMD remains underdiagnosed due to its rarity and the broad spectrum of clinical presentation [12]. Respiratory and hepatic dysfunctions are the primary causes of death in patients with ASMD type B, with no evidence of neurodegeneration [3,4,13]. In contrast, neurodegeneration along with respiratory failure and hepatic complications are the leading causes of death in ASMD type A/B [3,13]. ASMD significantly impacts daily functions and quality of life (QoL), leading to substantial social and economic burden, which highlights the necessity of an effective disease-modifying treatment [14,15].

Olipudase alfa (Xenpozyme^®^, Sanofi), an intravenous recombinant human ASM, is the first and only approved enzyme replacement therapy for treating non-central nervous system manifestations in ASMD across several countries, including the United States (US) [16], the European Union [17], Japan [18], and Brazil [19]. It was well tolerated, improved the predicted diffusing capacity of the lung for carbon monoxide (DL_CO_), decreased spleen and liver volumes, and addressed metabolic imbalances in adult and pediatric patients with ASMD type B or type A/B in placebo-controlled (ASCEND: NCT02004691) and single-arm (ASCEND-Peds: NCT02004704) trials, respectively [20,21,22].

Previous studies delineated the clinical characterization [11], burden of illness, predictors of disease-related morbidity, and healthcare resource utilization (HCRU) in ASMD across multiple countries including Brazil [12]. Recent retrospective studies on ASMD type B and type A/B have revealed high mortality rates in the US [23], Germany [24], and France [25], with limited evidence on the burden of illness of ASMD in Brazil. To address this evidence gap, a retrospective real-world study was conducted to evaluate the characteristics and clinical data of patients with ASMD type B and type A/B in Brazil.

## 2. Materials and Methods

### 2.1. Study Design and Participants

This observational, multicenter, retrospective study was conducted with pediatric, adolescent, and adult patients diagnosed with ASMD type B or type A/B (surviving and deceased) at Hospital de Clínicas de Porto Alegre (HCPA) in Brazil from January 1, 1986 to May 31, 2021. Patients with ASMD type A were excluded from the study. The demographics of all eligible patients were obtained from HCPA (full cohort). Treatment centers of eligible patients were subsequently identified, and medical chart records with retrievable data were extracted for alive-eligible patients from eight participating centers (subset cohort). However, medical records of deceased patients could not be retrieved retrospectively.

### 2.2. Data Collection

Data on demographics for the full cohort and developmental history, clinical findings/manifestations, hospitalization, and laboratory examination data for the subset cohort were extracted retrospectively at the index date, at each time point available from diagnosis, and at the last follow-up or death. The index date was defined as the date of symptom onset or diagnosis of ASMD, whichever occurred first during the study period. No diagnostic or disease monitoring procedures were mandated, and no investigational treatment was administered during the study. All data were collected using electronic case report forms.

### 2.3. Statistical Methods

All analyses were conducted as per the statistical analysis plan using SAS statistics software version 9.4 (SAS Institute Inc., Cary, NC, USA). No formal hypothesis testing and power calculation on the sample size were performed in this study; data were summarized using descriptive statistics. Continuous variables were presented as mean and standard deviation (SD) or median and interquartile range (IQR) at each longitudinal time point, while categorical variables were expressed as the number of observations and proportions. The proportion of patients with missing data and the corresponding reasons were summarized appropriately.

## 3. Results

### 3.1. Study Participants

**Figure 1:**
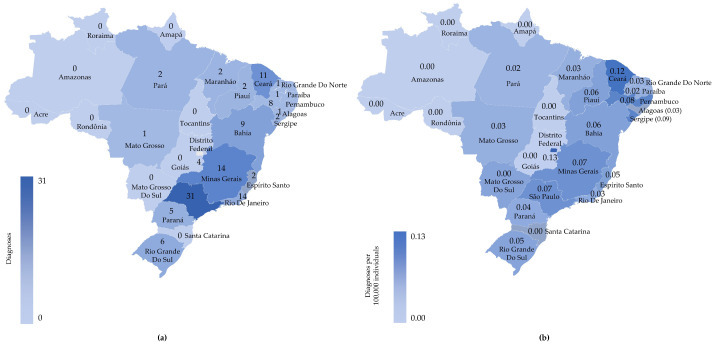
Characteristics of patients with ASMD in the full cohort; (**a**) geographic distribution of patients diagnosed with ASMD and (**b**) ASMD diagnoses per 100,000 individuals in Brazil (2021). Maps obtained from the platform Bing, ©Microsoft, OpenStreetMap. ASMD, acid sphingomyelinase deficiency.

A total of 124 patients with ASMD type B and type A/B (full cohort) were identified, and their demographic data (phenotype characterization, age, sex, and geographical distribution) were retrieved from HCPA (Figure S1). The majority of patients (75.8%, *n* = 94) were diagnosed with ASMD type B, while approximately one-fourth (24.2%, *n* = 30) had ASMD type A/B; half of the patients (50.4%, *n* = 60) were female. The demographic details of the patients are presented in Table S1. Of 124 patients, 108 had evidence on geographical location (Figure 1a). Notably, the State of Ceará and the Federal District (Distrito Federal) reported the highest rates (0.12 and 0.13 diagnoses per 100,000 individuals, respectively) of ASMD diagnoses in 2021 in Brazil (Figure 1b). However, the State of São Paulo recorded the highest number of patients (*n* = 31) with ASMD (Figure 1b).

### 3.2. Medical Chart Reviews for the Subset Cohort

Retrievable medical data and developmental history were evaluated for the subset cohort (*N* = 24); the majority of patients (87.5%, *n* = 21) were diagnosed with ASMD type B, while three patients (12.5%) had ASMD type A/B. The demographic characteristics and developmental history of these patients are presented in Table 1. No patient had a prior history of surgical splenectomy.

Patient distribution by age (<18 and ≥18 years) across phenotypes at symptom onset and diagnosis is illustrated in Figure S2. Evidently, the majority of patients (95.2%, *n* = 20 and 81.0%, *n* = 17) with ASMD type B and all patients (100.0%, *n* = 3 each) with ASMD type A/B were in the pediatric age group (<18 years) at symptom onset and diagnosis, respectively.

#### 3.2.1. Comorbidities

Half of the patients (50.0%, *n* = 12) at symptom onset or diagnosis and 66.7% of patients (*n* = 16) at the last follow-up or death had at least one comorbidity (Table S2).

#### 3.2.2. ASMD-Related Manifestations in the Subset Cohort

**Figure 2:**
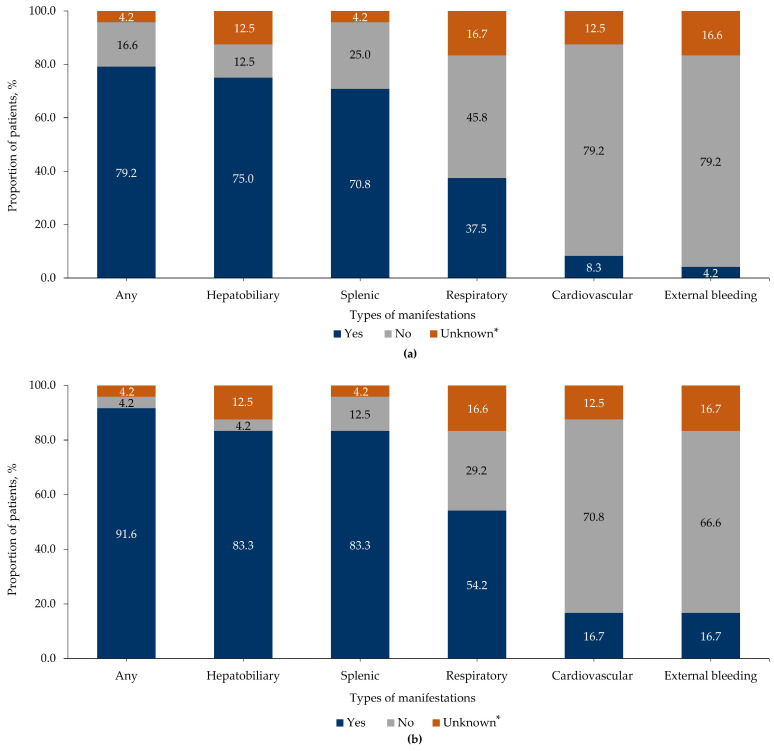
ASMD-related manifestations (**a**) at symptom onset or diagnosis and (**b**) at the last follow-up or death. The numbers in the bar graph represent the proportion of patients with available, unavailable, and unknown data on manifestations. ASMD, acid sphingomyelinase deficiency. * Unknown refers to missing data.

The majority of patients in this study showed manifestations within 1.5 years of symptom onset or diagnosis; 79.2% of patients (*n* = 19) presented with ASMD-related manifestations, which increased to 91.6% of patients (*n* = 22) at the last follow-up or death (Figure 2). Hepatobiliary and splenic manifestations were the most common clinical findings at symptom onset or diagnosis (75.0%, *n* = 18 and 70.8%, *n* = 17, respectively) and at the last follow-up or death (83.3%, *n* = 20 each) (Figure 2). The majority of patients (81.0%, *n* = 17 each) with ASMD type B and all patients (100.0%, *n* = 3 each) with ASMD type A/B exhibited hepatobiliary and splenic manifestations, followed by respiratory tract and cardiovascular manifestations, and external bleeding at the last follow-up or death (Table S3).

#### 3.2.3. Healthcare Resource Utilization in the Subset Cohort

At symptom onset or diagnosis, 33.3% of patients (*n* = 8) recorded at least one incidence of hospitalization or inpatient stay, and the incidence increased to 45.9% (*n* = 11) at the last follow-up or death (Table 2). However, none of the patients had any emergency room and outpatient visits at any time point. Notably, the incidence of hospitalization was higher in patients (100.0%, *n* = 3) with ASMD type A/B than that in patients (38.1%, *n* = 8) with ASMD type B at the last follow-up or death.

Overall, patients with ASMD had a median (IQR) inpatient stay of 6.5 (2.0–15.0) days at the hospital, whereas patients with type B and type A/B stayed in the hospital for 7.0 (2.0–29.0) days and 2.0 (0–7.0) days, respectively (Table 2), at the last follow-up or death.

#### 3.2.4. Laboratory Tests and Examination

**Figure 3:**
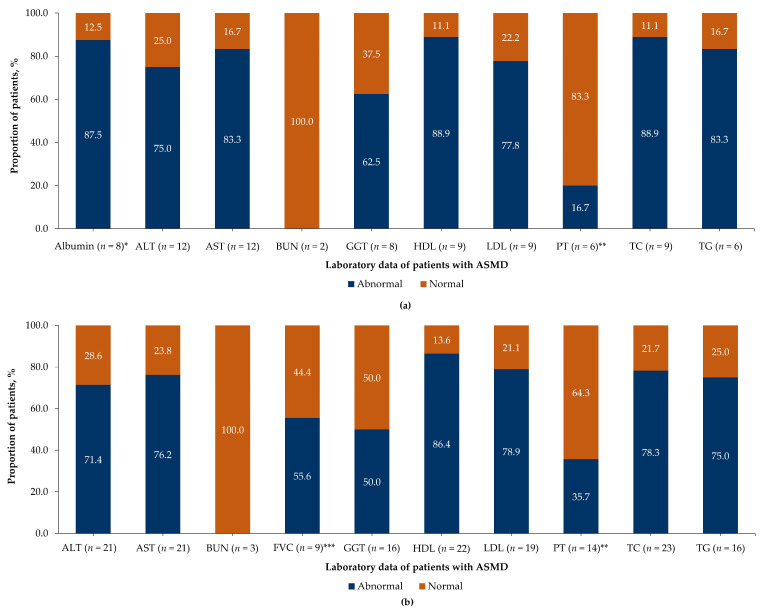
Laboratory observations of patients with ASMD (**a**) at diagnosis and (**b**) at the last follow-up or death. For (**a**,**b**), the numbers in the bar graph represent the proportions of patients with normal and abnormal laboratory data. The X-axis represents the proportions of patients with available data. ALT, alanine aminotransferase; ASMD, acid sphingomyelinase deficiency; AST, aspartate aminotransferase; BUN, blood urea nitrogen; FVC, forced vital capacity; GGT, gamma-glutamyl transferase; HDL, high-density lipoprotein; LDL, low-density lipoprotein; *n*, number of patients in each group; PT, prothrombin time; TC, total cholesterol; TG, triglyceride. * Albumin data were not available at the last follow-up or death. ** PT was expressed in seconds. *** FVC data were not available at diagnosis.

Elevated spleen (median [IQR]: 11.1 [8.5–16.8] multiples of normal) and liver (1.9 [1.9–2.3] multiples of normal) volumes were reported in 25.0% (*n* = 6) and 20.8% (*n* = 5) of patients with ASMD at the last follow-up or death, respectively, with no evidence of volume at diagnosis. The majority of patients with recorded liver function tests reported abnormal levels at diagnosis and the last follow-up or death, except for blood urea nitrogen, which was within the normal range for all patients (Figure 3a,b). Abnormal levels of high-density lipoprotein cholesterol were observed in 86.4% (*n* = 19/22), low-density lipoprotein cholesterol in 79.0% (*n* = 15/19), total cholesterol in 78.3% (*n* = 18/23), and triglycerides in 75.0% of patients (*n* = 12/16) at the last follow-up or death (Figure 3b). The proportion of patients with abnormal levels of LDL cholesterol and prothrombin time (seconds) increased from diagnosis (77.8% and 16.7%) to the last follow-up or death (78.9% and 35.7%) (Figure 3a,b).

The evidence of pulmonary function test was available for 4.2% of patients (*n* = 1) at symptom onset or diagnosis and 37.5% of patients (*n* = 9) at the last follow-up or death, with the records of predicted forced vital capacity (FVC) (Table S4) available for all patients. More than half of these patients (55.6%, *n* = 5/9) showed abnormal values for predicted FVC (≤85.0%) at the last follow-up or death (Figure 3b). The DL_CO_ data (%) were available for 12.5% of patients (*n* = 3/24) at the last follow-up or death; the predicted DL_CO_ value was ≤40.0% for two patients (66.7%) and >80.0% for one patient (Table S4).

#### 3.2.5. Mortality in the Subset Cohort

During the follow-up period, two patients (8.3%) with ASMD type A/B died, with mean (SD) age at death of 2.7 (1.9) years. One death was due to respiratory illness, while the cause of the second death was unspecified. The median (IQR) follow-up duration of ASMD was 11.3 (4.7–16.8) years from symptom onset or diagnosis, and the median (IQR) age at the last follow-up was 14.1 (5.8–19.3) years. However, for alive patients (*n* = 22), the median (IQR) age at the last follow-up was 14.8 (9.0–20.6) years.

## 4. Discussion

This retrospective study contributed to the current understanding of disease-related burden among patients with ASMD in Brazil. While a previous prospective study by McGovern et al. reported the natural history of chronic ASMD (ASMD type A/B and type B) and a longitudinal view of the disease spectrum in 59 patients across five countries, including Brazil (*n* = 13) [12,26], the present study shed light on the substantial morbidity associated with ASMD in Brazil. The study demonstrated a homogeneous distribution of both sexes, which is consistent with that of other studies on ASMD by McGovern et al. [12,26]. Evidence from the current study provided insights into patients’ prolonged diagnostic journey, leading to a delay in diagnosis of 8 years (>11 years in a few cases [*n* = 4]). A recent study indicated the challenges in accurately estimating the incidence of ASMD due to the inherent risk of underdiagnosis, underscoring the need for a diagnostic protocol [27]. Thus, the lack of timely diagnosis, specifically before 2000, might have accounted for the disease burden. However, recent advancements in molecular diagnostics and diagnostic protocols could facilitate an early detection of the disease [11,27]. The geographic distribution of patients in Brazil was heterogeneous due to differences in ethnicity [11], which highlighted the lack of disease awareness and underscored the requirement of additional diagnostic centers in Brazil to expedite the screening process.

The median age at symptom onset was lower (0.9 [0.1–4.2] years) in the subset cohort than in patients with ASMD in the US (3.9 [1.3–10.9] years; *N* = 110) [23], Germany (2.0 [1.0–5.0] years; *N* = 33) [24], and across multiple countries (2.0 [0.1–14.0] years, *N* = 59; Brazil, *n* = 13) [26], emphasizing the disease burden in the pediatric group, regardless of the disease type. The median (IQR) age recorded at the last follow-up or death also indicated a substantial burden of illness in the same group as patients were either children or adolescents at the last follow-up or had died, consistent with the reported high morbidity among pediatric patients with ASMD type B and type A/B in the US [23] and Germany [24].

Additionally, the presence of comorbidities and clinical manifestations contributed to the disease burden. The proportion of patients with comorbidities was higher at the last follow-up or death than at symptom onset or diagnosis. The most common disease-related clinical findings included splenic (83.3%), hepatobiliary (83.3%), and respiratory (54.2%) manifestations at the last follow-up or death, thus presenting a heterogeneous phenotypic spectrum and varying severity levels among patients with ASMD type B and type A/B in Brazil. Similarly to the results of this study, a retrospective study in the US showed that chronic liver dysfunctions, lung diseases, and myalgia were the most common observations at first presentation or diagnosis and the last follow-up or death [23]. Cassiman et al. reported similar disease-related morbidities, including splenomegaly (96.6%), hepatomegaly (91.4%), liver dysfunction (82.6%), and pulmonary disease (75.0%) in patients with ASMD type B and type A/B [13]. Liver and pulmonary diseases were reported as major morbidities in patients with ASMD type B [28].

Furthermore, in the present study, abnormal values of laboratory examinations (alanine aminotransferase [ALT], aspartate aminotransferase [AST], gamma-glutamyl transferase, and albumin) indicated the presence of liver and splenic manifestations in patients. These findings are consistent with the results of a previous study on the natural history of ASMD type B, which reported 75.0% and 68.0% of patients with abnormal ALT values and 65.0% and 69.0% of patients with abnormal AST values at the initial visit and the last follow-up, respectively [29]. Notably, dyslipidemia was reported in majority of the patients with ASMD in Brazil at diagnosis and at the last follow-up or death, consistent with the observations from a cross-sectional analysis by McGovern et al. [4,30].

Moreover, the proportion of patients experiencing respiratory manifestations at the last follow-up or death increased compared with that at symptom onset or diagnosis. Abnormal FVC levels were observed in 55.6% of patients and the mean (SD) predicted FVC at the last follow-up or death was 82.5% (19.0%, *n* = 9), indicating a restrictive pulmonary disease. Previously, McGovern et al. identified 47.0% of patients with abnormal FVC levels and a mean (range) value of 82.0% (48.0–118.0%) [12]. Additionally, this study reported two deaths in the subset cohort during the follow-up. Data on the mortality of deceased patients from the full cohort were unavailable or could not be retrieved. Thus, survival analysis could not be performed.

This study has several limitations. Data of only 124 patients were extracted from HCPA, with limited information on demographic characteristics, comorbidities, manifestations, and HCRU (*N* = 24). Thus, necessary analyses, including formal hypothesis testing and power calculation, could not be performed for this population. Data on patients’ genotype were not captured, which restricted the determination of genotype–phenotype correlation. Medical records of deceased patients could not be retrieved, restricting the mortality analyses and thereby limiting the representativeness of data. The generalizability of all ASMD-related clinical findings and associated manifestations in patients available for the subset cohort were limited considering the small sample size, and therefore, findings should be interpreted with caution. Also, lack of data regarding age at onset or diagnosis and at the last follow-up or death restricted the analyses to represent disease progression over time and the extent of organ involvement in patients. Disease burden could not be evaluated by comparing phenotypes because of the inadequate number of patients (*n* = 3) with ASMD type A/B. Moreover, data on patient-reported outcomes and QoL were not considered, thereby limiting insights into patients’ perspectives of the disease and its associated burden.

## 5. Conclusions

Overall, this study in Brazil demonstrates a substantial burden of illness associated with ASMD with respect to clinical manifestations, comorbidities, and disease-related complications, as well as a delay in diagnosis. This highlights the significance of disease awareness and underscores the need for an effective treatment to reduce the diagnostic journey of patients, mitigate the disease burden, and improve the lives of patients.

## Figures and Tables

**Table 1:** Demographic characteristics and developmental history of patients with ASMD in the subset cohort.

Demographic Characteristic and Developmental History	At Symptom Onset/Diagnosis			At Symptom Onset			At Diagnosis			At the Last Follow-Up/Death		
	Overall (*N* = 24)	ASMD Type B (*n* = 21)	ASMD Type A/B (*n* = 3)	Overall (*N* = 24)	ASMD Type B (*n* = 21)	ASMD Type A/B (*n* = 3)	Overall (*N* = 24)	ASMD Type B (*n* = 21)	ASMD Type A/B (*n* = 3)	Overall (*N* = 24)	ASMD Type B (*n* = 21)	ASMD Type A/B (*n* = 3)
**Age (years)**												
**Mean (SD)**	3.6 (7.4)	4.0 (7.8)	0.5 (0.4)	3.7 (7.3)	4.1 (7.6)	0.5 (0.4)	11.7 (15.3)	13.2 (15.8)	1.4 (0.3)	17.3 (14.7)	19.5 (14.5)	2.3 (1.5)
**Median (IQR)**	0.8 (0–4.2)	1.0 (0–4.3)	0.7 (0–0.8)	0.9 (0.1–4.2)	2.0 (0–4.3)	0.7 (0–0.8)	6.7 (1.9–11.3)	7.0 (3.0–11.8)	1.33 (1.2–1.7)	14.1 (5.8–19.3)	15.3 (11.5–20.6)	1.6 (1.3–4.1)
**Sex, *n* (%)**												
**Male**	10 (41.7)	8 (38.1)	2 (66.7)	NA								
**Female**	14 (58.3)	13 (61.9)	1 (33.3)									
**BMI (kg/m^2^)** ^^^												
***n* (%)**	4 (16.7)			4 (16.7)			12 (50.0)			19 (79.2)		
**Mean (SD)**	15.9 (1.9)			15.9 (1.9)			19.2 (5.6)			20.2 (5.0)		
**Median (IQR)**	15.9 (14.3–17.6)			15.9 (14.3–17.6)			17.7 (15.6–18.5)			18.5 (17.7–20.5)		
**BMI for age, Z-score** *^#^												
***n* (%)**	2 (8.3)			2 (8.3)			10 (41.7)			13 (54.1)		
**Mean (SD)**	−0.1 (1.6)			−0.1 (1.6)			−0.2 (0.9)			0 (1.0)		
**Median (IQR)**	−0.1 (−1.3 to 1.0)			−0.1 (−1.3 to 1.0)			0 (−1.27 to 0.6)			−0.2 (−0.6 to 0.8)		
**Weight (kg)** ^^^												
***n* (%)**	4 (16.7)			4 (16.7)			12 (50.0)			18 (75.0)		
**Mean (SD)**	17.5 (6.7)			17.5 (6.7)			31.4 (24.1)			38.4 (22.1)		
**Median (IQR)**	18.7 (12.4–22.7)			18.7 (12.4–22.7)			25.7 (14.2–34.9)			31.8 (19.4–51.9)		
**Weight for age, Z-score** ^#^												
***n* (%)**	4 (16.7)			4 (16.7)			10 (41.7)			13 (54.1)		
**Mean (SD)**	2.2 (3.1)			2.2 (3.1)			−1.8 (1.1)			−1.6 (1.5)		
**Median (IQR)**	1.3 (0.4–4.0)			1.3 (0.4–4.0)			−1.9 (−2.8 to −1.2)			−1.8 (−2.5 to −0.7)		
**Height (cm)** ^^^												
***n* (%)**	5 (20.8)			5 (20.8)			13 (54.2)			19 (79.2)		
**Mean (SD)**	115.9 (32.8)			116.0 (32.8)			123.1 (31.3)			135.2 (30.4)		
**Median (IQR)**	109.0 (105.0–118.0)			109.0 (105.0–118.0)			128.0 (110.0–146.0)			136.0 (104.4–163.0)		
**Height for age, Z-score** ^#^												
***n* (%)**	4 (16.7)			4 (16.7)			10 (41.7)			13 (54.1)		
**Mean (SD)**	3.8 (3.0)			3.8 (3.0)			−2.5 (1.2)			−2.6 (1.8)		
**Median (IQR)**	2.7 (1.9–5.6)			2.7 (1.9–5.6)			−2.7 (−3.1 to −1.5)			−2.5 (−4.1 to −1.2)		
**Diagnostic methods, *n* (%)**												
**Enzyme**	13 (54.2)	12 (57.1)	1 (33.3)	NA								
**Genetic and enzyme**	5 (20.8)	4 (19.0)	1 (33.3)									
**Genetic testing**	2 (8.3)	1 (4.8)	1 (33.3)									
**Other**	1 (4.2)	1 (4.8)	0									
**Unknown**	3 (12.5)	3 (14.3)	0									

ASMD, acid sphingomyelinase deficiency; BMI, body mass index; IQR, interquartile range; *N*, total number of patients; *n*, number of patients in the subgroup; NA, not available; SD, standard deviation. Weight was converted from pounds to kilograms using the following formula: weight in pounds divided by 2.205, and height was converted from inches to centimeters using the following formula: height in inches multiplied by 2.54. Height-for-age, weight-for-age, and BMI-for-age Z-scores were calculated with algorithms described by the World Health Organization Child Growth Standards. * One patient with ASMD type B in this cohort was >18 years old. ^^^ Data are presented irrespective of age. ^#^ Data are presented for patients < 18 years of age.

**Table 2:** Healthcare resource utilization in the subset cohort.

Parameters	At Symptom Onset or Diagnosis	At Symptom Onset	At Diagnosis	At the Last Follow-Up or Death			
	Overall (*N* = 24)	Overall (*N* = 24)	Overall (*N* = 24)	Overall (*N* = 24)	ASMD Type B (*n* = 21)	ASMD Type A/B (*n* = 3)	*p*-Value **
**At least one hospitalization/inpatient stay, *n* (%)**							
**No**	11 (45.9)	17 (70.8)	11 (45.9)	8 (33.3)	8 (38.1)	0	0.13
**Yes**	8 (33.3)	2 (8.4)	8 (33.3)	11 (45.9)	8 (38.1)	3 (100.0)	
**Unknown**	5 (20.8)	5 (20.8)	5 (20.8)	5 (20.8)	5 (23.8)	0	
**Time from symptom onset or diagnosis to first inpatient stay, years**							
**Mean (SD)**	NA			9.2 (16.3) *	12.9 (19.4) ^^^	1.7 (1.6)	0.36
**Median (IQR)**				1.7 (1.4–6.1) *	3.9 (1.5–17.1) ^^^	1.4 (0.2–3.4)	
**Inpatient stay, days**							
**Mean (SD)**	NA			14.5 (23.7)	19.4 (27.2)	3.0 (3.6)	0.34
**Median (IQR)**				6.5 (2.0–15.0)	7.0 (2.0–29.0)	2.0 (0–7.0)	
**Age at first inpatient stay, years**							
**Mean (SD)**	NA			9.8 (17.1) *	13.7 (20.4) ^^^	2.16 (1.7)	0.38
**Median (IQR)**				2.2 (1.4–7.1) *	4.7 (1.6–17.1) ^^^	1.4 (1.0–4.1)	

ASMD, acid sphingomyelinase deficiency; HCRU, healthcare resource utilization; IQR, interquartile range; LOS, length of stay; *N*, total number of patients; *n*, number of patients in the subgroup; NA, not available; SD, standard deviation. * Missing data, *N* = 15. ^ Missing data, *n* = 15. ** *p*-value is calculated for HCRU, comparing between proportion of patients with ASMD type B and type A/B. Inpatient stay was defined as a hospital stay ≥ 1 night (LOS > 1 day).

## Data Availability

Qualified researchers may request access to patient level data and related study documents including the clinical study report, study protocol with any amendments, blank case report form, statistical analysis plan, and dataset specifications. Patient level data will be anonymized, and study documents will be redacted to protect the privacy of our trial participants. Further details on Sanofi’s data sharing criteria, eligible studies, and process for requesting access can be found at: https://www.vivli.org/.

## Supplementary Materials

The following supporting information can be downloaded at: https://www.mdpi.com/article/10.3390/jcm15020589/s1, Table S1: Demographic details of the full cohort; Table S2: Comorbidities or medical conditions in addition to ASMD reported in the subset cohort; Table S3: ASMD-related clinical findings/manifestations of ASMD across phenotypes at the last follow-up or death; Table S4: Pulmonary function test of patients with ASMD in the subset cohort; Figure S1: Study design; Figure S2: Distribution of patients by age (a) at symptom onset and (b) at diagnosis across phenotypes.

## Abbreviations

The following abbreviations are used in this manuscript:

ALT	Alanine aminotransferase
ASM	Acid sphingomyelinase
ASMD	Acid sphingomyelinase deficiency
AST	Aspartate aminotransferase
BMI	Body mass index
BUN	Blood urea nitrogen
DL_CO_	Predicted diffusing capacity of the lung for carbon monoxide
FVC	Forced vital capacity
GGT	Gamma-glutamyl transferase
HCPA	Hospital de Clínicas de Porto Alegre
HCRU	Healthcare resource utilization
HDL	High-density lipoprotein
IQR	Interquartile range
LDL	Low-density lipoprotein
LOS	Length of stay
*N*	Total number of patients
*n*	Number of patients in each subgroup
NA	Not available/applicable
OMIM	Online Mendelian Inheritance in Man
PT	Prothrombin time
QoL	Quality of life
SD	Standard deviation
*SMPD1*	Sphingomyelin phosphodiesterase 1 gene
TC	Total cholesterol
TG	Triglyceride
US	United States
