# Supplementary material for: A Retrospective Chart Review Study on the Burden of Illness of Acid Sphingomyelinase Deficiency in Brazil
Source: J Clin Med. 2026 Jan 12;15(2):589. doi: 10.3390/jcm15020589 (PMC12841961; doi:10.3390/jcm15020589)
Supplement: Supplementary file 1 [file jcm-15-00589-s001.zip › Supplementary Figures.pdf]

Journal of
Clinical Medicine

MDPI

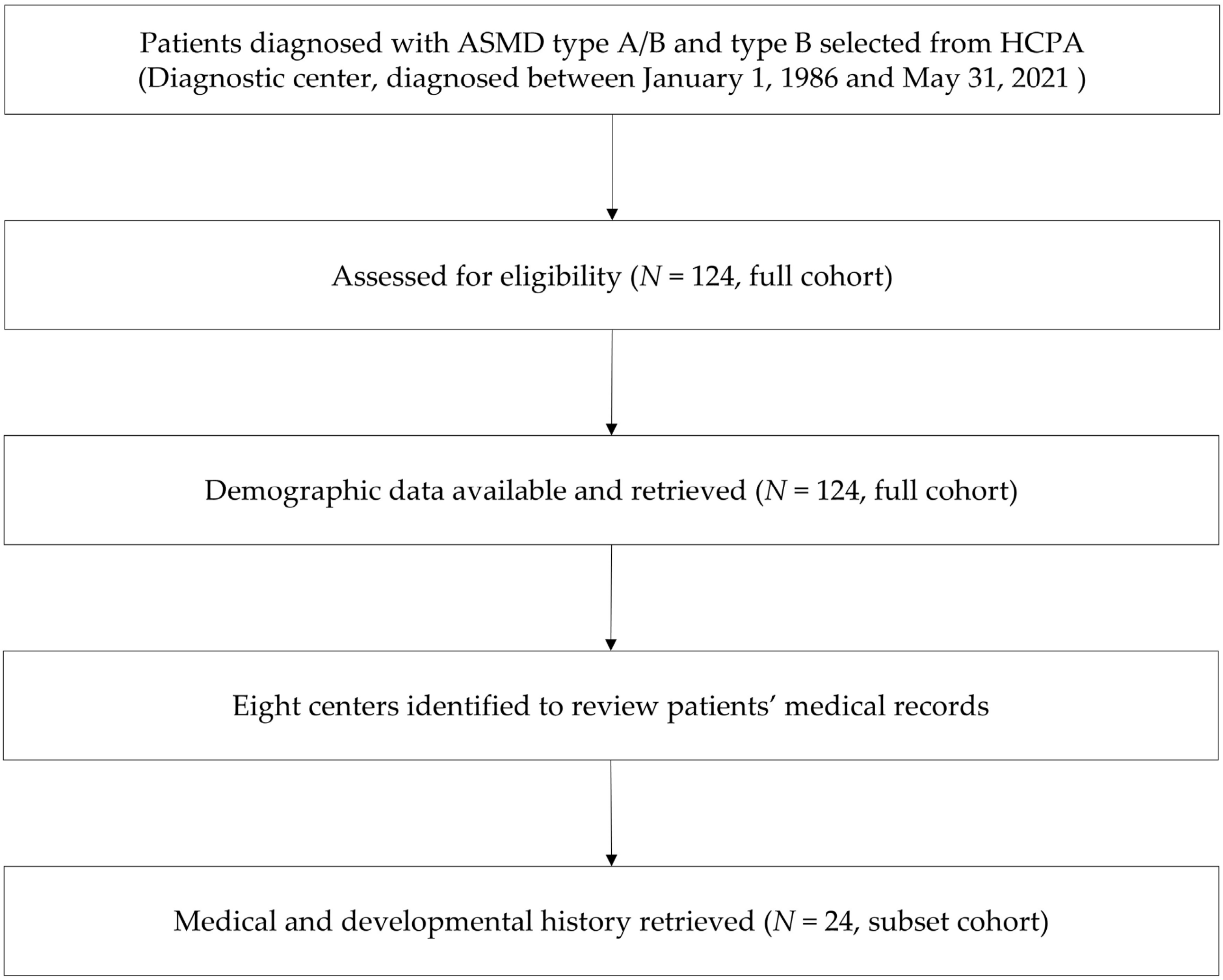

Figure S1. Study design

ASMD, acid sphingomyelinase deficiency; HCPA, Hospital de Clínicas de Porto Alegre, *N*, total number of patients in full cohort and subset cohort.

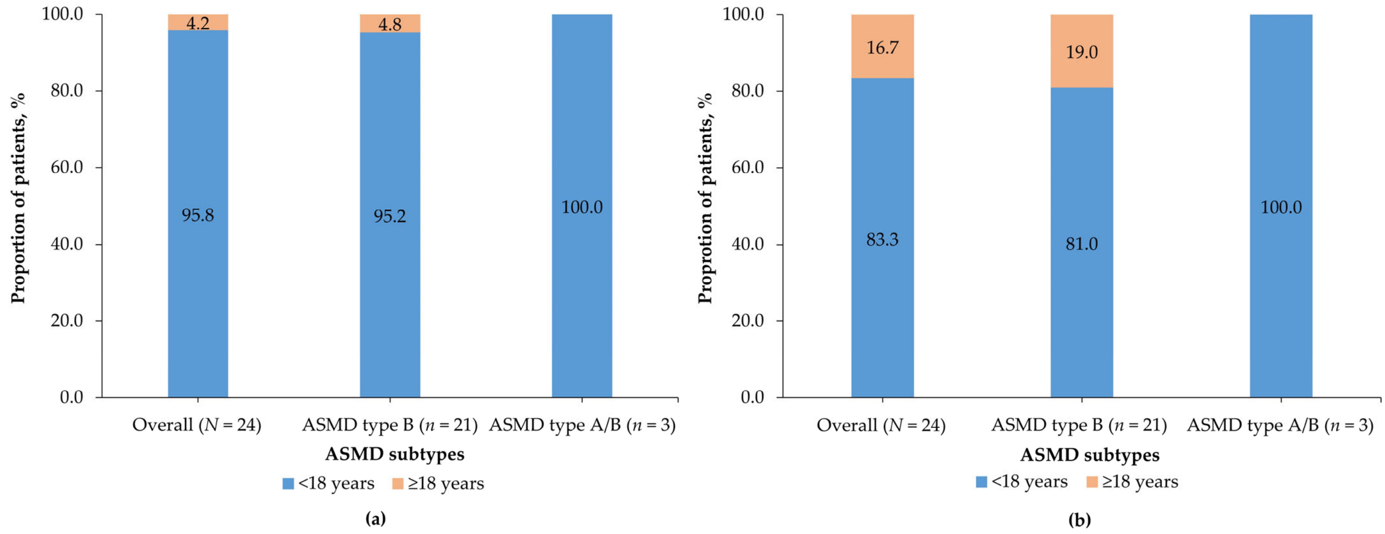

Figure S2. Distribution of patients by age (**a**) at symptom onset and (**b**) at diagnosis across phenotypes.

Numbers in the bar graph represent the percentage of patients <18 years and ≥18 years of age. ASMD, acid sphingomyelinase deficiency; *n*, number of patients in the subgroup.
