# Supplementary material for: A Retrospective Chart Review Study on the Burden of Illness of Acid Sphingomyelinase Deficiency in Brazil
Source: J Clin Med. 2026 Jan 12;15(2):589. doi: 10.3390/jcm15020589 (PMC12841961; doi:10.3390/jcm15020589)
Supplement: Supplementary file 1 [file jcm-15-00589-s001.zip › Table S1.pdf]

Supplementary material

Table S1. Demographic details of the full cohort

Demographic characteristics	Full cohort (*N* = 124)
**Phenotype**	
**ASMD type B**	94 (75.8%)
**ASMD type A/B**	30 (24.2%)
**Sex ***	
**Female**	60 (50.4%)
**Male**	59 (49.6%)
**Age at diagnosis (years) ^**	
**Mean (SD)**	16.4 (12.9)
**Median (IQR)**	10.0 (3.6–19.9)

ASMD, acid sphingomyelinase deficiency; IQR, interquartile range; *N*, total number of patients; SD, standard deviation. * Patients with missing data (*n* = 5). ^ Patients with missing data (*n* = 30).
