# Supplementary material for: A Retrospective Chart Review Study on the Burden of Illness of Acid Sphingomyelinase Deficiency in Brazil
Source: J Clin Med. 2026 Jan 12;15(2):589. doi: 10.3390/jcm15020589 (PMC12841961; doi:10.3390/jcm15020589)
Supplement: Supplementary file 1 [file jcm-15-00589-s001.zip › Table S2.pdf]

J. Clin. Med. **2025**, *15*, 589

Supplementary material

Table S2. Comorbidities or medical conditions in addition to ASMD reported in the subset cohort

Types of comorbidities	At symptom onset or diagnosis	At symptom onset	At diagnosis	At the last follow-up or death
**At least one comorbidity, *n* (%)**				
No	7 (29.2)	15 (62.5)	7 (29.2)	3 (12.5)
Yes	12 (50.0)	4 (16.7)	12 (50.0)	16 (66.7)
Unknown #	5 (20.8)	5 (20.8)	5 (20.8)	5 (20.8)
**Myocardial infarction, *n* (%)**				
No	19 (79.2)	19 (79.2)	19 (79.2)	19 (79.2)
Yes	0	0	0	0
Unknown #	5 (20.8)	5 (20.8)	5 (20.8)	5 (20.8)
**Cerebrovascular accident, *n* (%)**				
No	19 (79.2)	19 (79.2)	19 (79.2)	19 (79.2)
Yes	0	0	0	0
Unknown #	5 (20.8)	5 (20.8)	5 (20.8)	5 (20.8)
**Cardiac valvular abnormality, *n* (%)**				
No	19 (79.2)	19 (79.2)	19 (79.2)	19 (79.2)
Yes	0	0	0	0
Unknown #	5 (20.8)	5 (20.8)	5 (20.8)	5 (20.8)
**Hemorrhagic diathesis or abnormal bleeding, *n* (%)**				
No	19 (79.2)	19 (79.2)	19 (79.2)	18 (75.0)
Yes	0	0	0	1 (4.2)
Unknown #	5 (20.8)	5 (20.8)	5 (20.8)	5 (20.8)
**Hematologic malignancies (e.g., thrombocytopenia, anemia, etc.), *n* (%)**				
No	17 (70.8)	18 (75.0)	17 (70.8)	16 (66.7)
Yes	2 (8.3)	1 (4.2)	2 (8.3)	3 (12.5)
Unknown #	5 (20.8)	5 (20.8)	5 (20.8)	5 (20.8)
**Lung diseases (e.g., interstitial lung disease, etc.), *n* (%)**				
No	18 (75.0)	19 (79.2)	18 (75.0)	17 (70.8)
Yes	1 (4.2)	0	1 (4.2)	2 (8.3)
Unknown #	5 (20.8)	5 (20.8)	5 (20.8)	5 (20.8)
**Asthma, *n* (%)**				
No	17 (70.8)	19 (79.2)	17 (70.8)	15 (62.5)
Yes	2 (8.3)	0	2 (8.3)	4 (16.7)
Unknown #	5 (20.8)	5 (20.8)	5 (20.8)	5 (20.8)
**Tobacco use, *n* (%)**				
No	19 (79.2)	19 (79.2)	19 (79.2)	19 (79.2)
Yes	0	0	0	0
Unknown #	5 (20.8)	5 (20.8)	5 (20.8)	5 (20.8)
**Hepatitis C, *n* (%)**				
No	18 (75.0)	19 (79.2)	18 (75.0)	18 (75.0)
Yes	1 (4.2)	0	1 (4.2)	1 (4.2)
Unknown #	5 (20.8)	5 (20.8)	5 (20.8)	5 (20.8)
**Chronic liver dysfunction, *n* (%)**				

No	18 (75.0)	19 (79.2)	18 (75.0)	18 (75.0)
Yes	1 (4.2)	0	1 (4.2)	1 (4.2)
Unknown #	5 (20.8)	5 (20.8)	5 (20.8)	5 (20.8)
**Congestive heart failure, *n* (%)**				
No	19 (79.2)	19 (79.2)	19 (79.2)	19 (79.2)
Yes	0	0	0	0
Unknown #	5 (20.8)	5 (20.8)	5 (20.8)	5 (20.8)
**Myalgia, *n* (%)**				
No	19 (79.2)	19 (79.2)	19 (79.2)	19 (79.2)
Yes	0	0	0	0
Unknown#	5 (20.8)	5 (20.8)	5 (20.8)	5 (20.8)
**Other comorbidities, *n* (%)**				
No	12 (50.0)	16 (66.7)	12 (50.0)	11 (45.8)
Yes	7 * (29.2)	3 (12.5)	7 (29.2)	8 ** (33.3)
Unknown #	5 (20.8)	5 (20.8)	5 (20.8)	5 (20.8)

Comorbidities at symptom onset or diagnosis were defined as any record of a comorbidity before or at symptom onset or diagnosis dates (maximum date between both). Comorbidities at the last follow-up or death were defined as any record of a comorbidity before or at the last follow-up or death. ASMD, acid sphingomyelinase deficiency; *n*, number of patients in the subgroup. # Unknown refers to missing data. *'Other' comorbidities in patients (*n* = 7) at symptom onset or diagnosis included hepatosplenomegaly, dyslipidemia, splenomegaly, dwarfism, lipid profile changes, dyspnea, obesity, liver complications; some patients had >1 'other' comorbidities. **'Other' comorbidities in patients (*n* = 8) at the last follow-up or death included hepatosplenomegaly, dyslipidemia, splenomegaly, dwarfism, lipid profile changes, dyspnea, obesity, liver complications, chronic pangastritis, lymphomegaly, hypovitaminosis D, septic teeth, elevated transaminases, thrombocytopenia, and epigastric abdominal pain; some patients reported >1 'other' comorbidities.
