# Supplementary material for: A Retrospective Chart Review Study on the Burden of Illness of Acid Sphingomyelinase Deficiency in Brazil
Source: J Clin Med. 2026 Jan 12;15(2):589. doi: 10.3390/jcm15020589 (PMC12841961; doi:10.3390/jcm15020589)
Supplement: Supplementary file 1 [file jcm-15-00589-s001.zip › Table S3.pdf]

J. Clin. Med. **2025**, *15*, 589

Supplementary material

Table S3. ASMD-related clinical findings/manifestations of ASMD across phenotypes at the last follow-up or death

Types of manifestations	Overall (*N* = 24)	ASMD type B (*n* = 21)	ASMD type A/B (*n* = 3)	*p*-value **
**At least one manifestation, *n* (%)**				
No	1 (4.2)	1 (4.8)	0	0.24
Yes	22 (91.6)	19 (90.4)	3 (100.0)	
Unknown *	1 (4.2)	1 (4.8)	0	
**Hepatobiliary, *n* (%)**				
No	1 (4.2)	1 (4.8)	0	0.71
Yes	20 (83.3)	17 (81.0)	3 (100)	
Unknown *	3 (12.5)	3 (14.2)	0	
**Splenic, *n* (%)**				
No	3 (12.5)	3 (14.3)	0	0.68
Yes	20 (83.3)	17 (81.0)	3 (100)	
Unknown *	1 (4.2)	1 (4.7)	0	
**Respiratory tract, *n* (%)**				
No	7 (29.2)	6 (28.6)	1 (33.3)	0.71
Yes	13 (54.2)	11 (52.4)	2 (66.7)	
Unknown *	4 (16.7)	4 (19.0)	0	
**Cardiovascular, *n* (%)**				
No	17 (70.8)	15 (71.4)	2 (66.7)	0.60
Yes	4 (16.7)	3 (14.3)	1 (33.3)	
Unknown *	3 (12.5)	3 (14.3)	0	
**External bleeding, *n* (%)**				
No	16 (66.6)	13 (61.9)	3 (100)	0.42
Yes	4 (16.7)	4 (19.0)	0	
Unknown *	4 (16.7)	4 (19.0)	0	

ASMD, acid sphingomyelinase deficiency; *N*, number of patients in the group; *n*, number of patients in the subgroup. * Unknown refers to missing data. ** *p*-value is calculated for ASMD-related clinical findings/manifestations, comparing between proportion of patients with ASMD type B and type A/B.
