# Supplementary material for: A Retrospective Chart Review Study on the Burden of Illness of Acid Sphingomyelinase Deficiency in Brazil
Source: J Clin Med. 2026 Jan 12;15(2):589. doi: 10.3390/jcm15020589 (PMC12841961; doi:10.3390/jcm15020589)
Supplement: Supplementary file 1 [file jcm-15-00589-s001.zip › Table S4.pdf]

Journal of Clinical Medicine

MDPI

Supplementary material

Table S4. Pulmonary function test of patients with ASMD in the subset cohort

<table>
<tr><th>Parameters</th><th>At symptom onset or diagnosis, whichever was earlier</th><th>At symptom onset</th><th>At diagnosis</th><th>At the last follow-up or death</th></tr>
<tr><td colspan="5">PFT, n (%)</td></tr>
<tr><td colspan="5">Any pulmonary test (DL_{CO} or FVC)</td></tr>
<tr><td>No</td><td>23 (95.8)</td><td>24 (100.0)</td><td>23 (95.8)</td><td>15 (62.5)</td></tr>
<tr><td>Yes</td><td>1 (4.2)</td><td>0</td><td>1 (4.2)</td><td>9 (37.5)</td></tr>
<tr><td>Predicted DL_{CO} only</td><td rowspan="2" colspan="3">0</td><td>0</td></tr>
<tr><td>Predicted DL_{CO} and FVC *</td><td>3 (33.3)</td></tr>
<tr><td>FVC only</td><td>1 (100.0)</td><td>0</td><td>1 (100.0)</td><td>6 (66.7)</td></tr>
<tr><td colspan="5">Predicted DL_{CO} (%) #</td></tr>
<tr><td>n</td><td rowspan="5" colspan="3">NA</td><td>3 (12.5)</td></tr>
<tr><td>Mean (SD)</td><td>42.7 (36.0)</td></tr>
<tr><td>Median (IQR)</td><td>40.0 (8.2–80.0)</td></tr>
<tr><td>≤40</td><td>2 (66.7)</td></tr>
<tr><td>>40</td><td>1 (33.3) ^</td></tr>
<tr><td colspan="5">Predicted FVC (%), as recorded</td></tr>
<tr><td>n</td><td>1 (4.2)</td><td rowspan="3">NA</td><td>1 (4.2)</td><td>9 (37.5)</td></tr>
<tr><td>Mean (SD)</td><td>67.0 (0)</td><td>67.0 (0)</td><td>82.5 (19.0)</td></tr>
<tr><td>Median (IQR)</td><td>67.0 (67.0–67.0)</td><td>67.0 (67.0–67.0)</td><td>78.0 (68.0–102.0)</td></tr>
<tr><td colspan="5">Predicted FVC (L), as recorded</td></tr>
<tr><td>Mean (SD)</td><td>1.4 (0)</td><td>NA</td><td>1.4 (0)</td><td>2.5 (1.1)</td></tr>
</table>

**Median (IQR)**	1.4 (1.4–1.4)		1.4 (1.4–1.4)	2.0 (1.8–3.0)
**Abnormal (≤85% of predicted), *n* (%)**	1 (100.0)		1 (100.0)	5 (55.6)
**Normal (>85% of predicted), *n* (%)**		NA		4 (44.4)

Predicted DL_{CO} value at the last follow-up or death was the last available value from diagnosis, symptom onset, or the last follow-up. Predicted DL_{CO} value at the end of follow-up or death is the last available value from diagnosis, symptom onset or last follow-up. ASMD, acid sphingomyelinase deficiency; DL_{CO}, diffusing capacity of the lung for carbon monoxide; FVC, forced vital capacity; IQR, interquartile range; L, liter; *n*, number of patients in the subgroup; NA, not available/applicable; PFT, pulmonary function test; SD, standard deviation. * FVC data were available for patients only at the last follow-up or death. [#] A regression analysis of the predicted DL_{CO} categorical variable was not feasible because of the small sample size per category. [^] This patient had a predicted DL_{CO} of >80%.
